# Neurodevelopmental toxicity of prenatal polychlorinated biphenyls (PCBs) by chemical structure and activity: a birth cohort study

**DOI:** 10.1186/1476-069X-9-51

**Published:** 2010-08-23

**Authors:** Hye-Youn Park, Irva Hertz-Picciotto, Eva Sovcikova, Anton Kocan, Beata Drobna, Tomas Trnovec

**Affiliations:** 1 Divisions of Epidemiology, and of Environmental and Occupational Health, Department of Public Health Sciences, School of Medicine, University of California, Davis, 95616, USA; 2 Department of Toxic Organic Pollutants, Slovak Medical University, Bratislava, Slovakia

## Abstract

**Background:**

Polychlorinated biphenyls (PCBs) are ubiquitous environmental toxins. Although there is growing evidence to support an association between PCBs and deficits of neurodevelopment, the specific mechanisms are not well understood. The potentially different roles of specific PCB groups defined by chemical structures or hormonal activities e.g., dioxin-like, non-dioxin like, or anti-estrogenic PCBs, remain unclear. Our objective was to examine the association between prenatal exposure to defined subsets of PCBs and neurodevelopment in a cohort of infants in eastern Slovakia enrolled at birth in 2002-2004.

**Methods:**

Maternal and cord serum samples were collected at delivery, and analyzed for PCBs using high-resolution gas chromatography. The Bayley Scales of Infant Development -II (BSID) were administered at 16 months of age to over 750 children who also had prenatal PCB measurements.

**Results:**

Based on final multivariate-adjusted linear regression model, maternal mono-ortho-substituted PCBs were significantly associated with lower scores on both the psychomotor (PDI) and mental development indices (MDI). Also a significant association between cord mono-ortho-substituted PCBs and reduced PDI was observed, but the association with MDI was marginal (*p *= 0.05). Anti-estrogenic and di-ortho-substituted PCBs did not show any statistically significant association with cognitive scores, but a suggestive association between di-ortho-substituted PCBs measured in cord serum and poorer PDI was observed.

**Conclusion:**

Children with higher prenatal mono-ortho-substituted PCB exposures performed more poorly on the Bayley Scales. Evidence from this and other studies suggests that prenatal dioxin-like PCB exposure, including mono-ortho congeners, may interfere with brain development *in utero*. Non-dioxin-like di-ortho-substituted PCBs require further investigation.

## Background

Polychlorinated biphenyls (PCBs) are ubiquitous environmental toxins. Improper disposal has been a major source of environmental contamination. Their production and use were banned in most industrialized countries in the late 1970s because of toxic effects in wildlife [1,2].

PCBs have been shown to have toxic effects on various organs including tissues of the nervous, reproductive, and immunologic systems [3-7]. Although there is growing evidence from *in vivo *and *in vitro *studies to support the hypothesis of adverse effects of PCBs on neurodevelopment [6-9], the mechanisms are not well understood. Additionally, various epidemiological studies have found an association between PCB exposure and decreased cognitive development [10-15].

The most well-known mechanism related to adverse health effects such as immune suppression, hepatotoxicity, and thymic atrophy is aryl hydrocarbon (Ah) receptor-mediated pathways for dioxin- like PCBs [16,17]. Since non-dioxin-like PCBs have shown low affinity for the Ah receptor [18], they have been regarded as potentially less toxic. However, neurotoxicity [19], carcinogenicity [20,21], and changes in hormones [22] have also been described as resulting from non-dioxin-like PCBs.

Alteration of sex-steroid hormone homeostasis by PCBs has been studied, and estrogen-like activity of PCBs was reported to change brain dopamine (DA) concentration [23,24] or aromatase activity [25]. Gonadal hormones such as estrogens can alter the structure and function of the hippocampus, which is critical for spatial and declarative learning and memory, and they modulate neural circuits by interacting with various neurotransmitters [26]. Besides playing a major role in sex differences, including sexual dimorphic behaviors, they modulate hypoxia-induced injury of neurons in the hippocampus [27] and influence synaptic patterning [28]. Estradiol protects against free radicals and regulates apoptosis [29], and these effects might depend on brain regions and timing of exposure. When bound to estrogen receptors, the complex regulates transcription[30]. PCBs have been proposed to affect brain development through an estrogen-receptor mediated pathway [31]. Specific steroidal pathways that might mediate PCB effects on cognitive and motor development have yet to be determined.

Previously, several epidemiological studies have evaluated the effects of PCBs on neurodevelopment. If only the Bayley Scales of Infant Development (BSID) assessment is considered, findings across studies are inconsistent. A Michigan study in a fishing community [32] and a study from 12 sites across the United States [33] found no association between BSID scores at 8 months and prenatal PCB exposure. In contrast, studies from North Carolina [34,35] and the Netherlands [14] observed prenatal PCB exposure to be associated with lower psychomotor development index (PDI) scores at 3, 6, 12, and 24 months whereas a study in Germany [15] found PCBs in breast milk, used as a surrogate measure for prenatal exposure, to be associated with decreased mental development index (MDI), but not with psychomotor development index (PDI), at 7 months of age. Several previous studies that used other instruments than the Bayley scales at different ages reported that PCBs were associated with detrimental effects on children's neurodevelopment [10,12,13,36,37].

Because of the lengthy half-lives of PCBs, any measurement in serum represents an accumulation of exposure over a period of years. These compounds circulate in the serum lipids and cross the placenta, albeit less efficiently than their hydroxy-metabolites [38]; nonetheless, the consistency of cord:maternal ratios across exposure levels provides the rationale for use of maternal concentrations as a surrogate for fetal exposures. The present study investigated associations between prenatal PCB exposures, measured from maternal and cord sera, characterized by their chemical structures and hormonal activities, and neurodevelopment measured by BSID among 16-month-old infants in eastern Slovakia.

## Methods

### Study population

Mother/infant pairs for a birth cohort were recruited at delivery between 2002 and 2004 from two districts in eastern Slovakia--Michalovce with high PCB contamination from a chemical manufacturing plant, and Svidnik located around 70 kilometers to the northwest, with lower levels of PCBs. Each district has only one hospital, and the vast majority of births from these regions occur in these hospitals. For eligibility criteria, we excluded women with more than four previous births, who were less than 18 years old at the time of their child's birth, or who had resided fewer than five years in their district. Mothers with a major health condition such as cancer or mental illness and infants who had severe birth defects were also excluded.

Of the 1134 eligible participants, maternal PCB measurements were determined in 1076 (blood samples were not collected for the remaining 58), and because of limited funding, cord PCBs were measured in 469. The cord measurements were conducted on all samples as they came into the laboratory until funding ran out, and they are a sub-set of maternal serum samples. Of those with maternal PCBs, data from 316 subjects were excluded owing to missing BSID scores mostly resulting from loss-to-follow-up (n = 311), or to missing lipid measurements (n = 5), leaving n = 760. From the 469 cord PCB measures, infants without BSID scores (n = 156) or lipid values (n = 55) were excluded, leaving n = 258. Therefore, for the current statistical analysis of neurodevelopment, we included those with BSID scores and maternal serum PCB measurements (n = 760) or with BSID scores and cord serum PCB measurements (n = 258). This study was approved by Institutional Review Boards of the University of California at Davis, USA (UC Davis) and the Slovak Medical University (SMU) in Bratislava, Slovakia. Written informed consent was obtained from all participants, and consent was given by the parents for infants to participate in the study.

### Specimen collection

All tubes of maternal and cord sera were refrigerated at 5 -10°C immediately after collection of blood at delivery. Serum was isolated by centrifugation (15 min at 3000 RPM) in each local hospital and placed into a freezer (-18°C). These specimens were then transported to the SMU in thermo boxes with cooling cartridges to prevent thawing, and after aliquoting 0.2 ml for lipid measurement, stored at -18°C until PCB analysis was performed.

### Laboratory measurements

The concentrations of 15 PCB congeners (PCB IUPAC #28, #52, #101, #105, #114, #118, #123, #138, #153, #156, #157, #167, #170, #180, and #189) were determined in the maternal and cord serum samples by high-resolution gas chromatography with electron capture detection [39,40]. This choice was based on the capability of gas chromatographic (GC) electron capture detection (ECD) to provide sufficient response for the so-called indicator PCB and dioxin-like PCB congeners. for example, the non-ortho substituted dioxin-like PCBs (#77, 81, 126, and 169) were not determinable by means of ECD when 4 mL or less of serum is available, as results would all be below the limits of detection (LOD). Additionally, PCB levels in babies are lower than those in adults and, moreover, the collected volume of serum was often low. As a consequence, only a handful of PCB congeners (#118, 138, 153, 156, 170, and 180) out of the 15 had most values above their LODs. Fitting models to poorly characterized exposure distributions is likely to be misleading, as would be the case if most sample results are < LOD. We focused our statistical analyses on the 6 congeners for which a high proportion of the samples was > LOD.

For analysis of PCBs, PCB #174 as an extraction standard was added to the blood serum before the analytes were isolated using solid phase extraction. The solid-phase extraction (SPE) extract was concentrated and then cleaned by passing through a Florisil-H_2_SO_4_/silica gel column. The eluate was then evaporated to a small volume, and PCB #103 was added as a syringe standard to correct the final volume of samples before GC injection. An aliquot of the mixture was injected and analyzed on a chromatography system (HP 5890, Hewlett-Packard, Palo Alto, CA) equipped with a Ni-63 electron capture detector using a 60m DBP-5 capillary column(J&W Scientific, Folsom, MA). Quantification was based on the calibration curve generated by authentic PCB standard solutions at five different concentration levels.

Quality control activities consisted of analyses of samples in batches of 10 simultaneously with a blank sample and in-house reference material (spiked porcine serum). Response for a particular congener had to be in the range of 90-110% using the concentration of the middle point of the calibration curves for that congener. The limit of detection for each analyte was determined as the mean of background noise plus three standard deviations from five reagent blank samples. The detailed methods for analysis have been described previously [39,40].

The Department of Toxic Organic Pollutants at the SMU performed the laboratory analyses. This accredited laboratory has annually participated successfully in an inter-laboratory comparison program organized by the German External Quality Assessment Scheme (G-EQUAS) for Analysis in Biological Materials at the Friedrich-Alexander-University, Erlagen-Nuremberg as well as the WHO-Coordinated Interlaboratory Quality Assessment of Levels of PCBs, PCDDs and PCDFs in Human Milk and Blood Plasma [35].

### Lipids measurement

Total serum lipids (TL) were estimated using enzymatic summation method [41]. Serum total cholesterol (TC) and triglyceride (TG) were measured using a DuPont aca III analyzer and cholesterol oxidase without cholesterol esterase was used to detect free cholesterol (FC). The method by Takayama et al.[42] was used to determine serum choline-containing phospholipids (PL). Total serum lipids were calculated from the formula TL = 1.677*(TC-FC) +FC+TG+PL. The lipids were measured at a biochemical laboratory accredited by the Slovak National Accreditation Service located at the Ministry of Defense Military hospital in Bratislava.

### Neurodevelopmental assessment

The BSID-II [43] are age standardized scales for 1- 42 months used widely in screening in clinical practice and for research studies of factors influencing development. Choosing a specific neurodevelopment assessment tool is complicated, in particular, for non-English speaking countries. At 16 months of age, this was the best general tool available with measures of both cognitive and motor skills, two key indicators of development in the second year of life, and the fact that the previous version (I) of BSID had been standardized and used in Slovakia was also an argument in its favor. Because BSID (II) had been used in previous studies of PCBs, this choice ensured comparability. The BSID (II) was administered by one local psychologist in each district at the study's 16-month follow-up visit. The two psychologists were trained by one of the investigators (ES) and a scientific advisory board member, both of whom are experienced psychologists.

We used the two scales of the BSID, the mental development index (MDI) and psychomotor development index (PDI) for the constructs of cognitive and motor development, respectively. The standardized MDI and PDI scores of BSID were calculated from raw scores based on child's actual age and are reported as the outcomes of interest. We videotaped 34 children in each district using a fixed camera and the tapes were reviewed by the psychologist in the other district to evaluate inter-rater reliability.

### Covariate measures

Major data sources for covariates for this analysis were an interview with the mother conducted by trained staff during the 5-day hospital stay after delivery, and the newborn medical record. In addition to the collection of socio-demographic and lifestyle factors, the Raven's Progressive Matrices [44], a non-verbal intelligence test, was administered to mothers after delivery. Additionally, the Home Observation for Measurement of the Environment (HOME) [45], which assesses the quality and quantity of stimulation given to a children in the home environment, was administered at the clinics during the 16-month follow-up visit. Because the HOME was administered at the clinics instead of the homes, we deleted 3 questions from the original version that were based on direct observations about play materials in the home. In a birth cohort study from Dusseldorf, scores from the modified version were highly correlated with scores from the full scale (Gerhard Winneke and Jens Walkowiak, Heinrich-Heine-University Dusseldorf, personal communication, 2003).

### Categorization of exposure variables

PCBs were categorized into different groups by their chemical structures (e.g., dioxin-like mono-ortho PCBs or non-dioxin-like di-ortho PCBs) or their estrogenic or anti-estrogenic properties based on the published literature [22,46-54]. In our analyses, we included six of the most abundant PCB congeners (PCB IUPAC #118, #138, #153, #156, #170, and #180). For the values below limits of detection (LOD), we imputed by taking the LOD value divided by the square root of 2 for congeners that had fewer than 20% of samples below the LOD [55,56] and the LOD divided by 2 if >20% of samples fell below the LOD (e.g., cord concentrations of PCB #118, and #156, which had 41.5%, and 40.0%, respectively, below the LOD)

The two mono-ortho dioxin-like PCBs we measured were #118 and #156 [17]. For non-dioxin-like di-ortho PCBs, PCB #138, #153, #170, and #180 were included [21]. PCB #138, #156, #170, and #180 were categorized as anti-estrogenic. However, estrogenic PCB levels (#28, #52, and #101) were low, with values for several congeners mostly below the LOD (range: 60-85%) and hence were not assessed for associations with neurodevelopmental scores in this study. These congener measurements would be expected to largely represent noise. Moreover, another investigation failed to find estrogenic activity of these congeners [57]. Because of the reports of mixed activity [22,46-50], PCB #105, #118, and #153 were excluded as anti-estrogenic PCBs for the analysis.

### Data analysis

PCB concentrations were obtained on a wet weight basis (ng/ml), and then adjusted for serum lipids (ng/mg total lipid). As distributions of chemical concentrations were skewed (Shapiro-Wilk test: *p *< 0.0001), lipid adjusted PCB values were natural log- transformed.

As a first step in the data analysis, we examined the crude association between PCBs and neurodevelopment. To screen for confounders, we did bivariate analyses regressing MDI scores, PDI scores, or PCBs on each covariate (e.g., maternal age, education, smoking and alcohol consumption, parity, ethnicity, gestational age, birth weight, child's sex, and maternal illness history), and examined these variables in a directed acyclic graph [58].

Then multiple linear regression models were developed with a broad inclusion of all covariates showing an association with the PDI or MDI of *p *< 0.3. We used a backward elimination approach and a change-in-estimate of 10% as the criterion [59] for deciding which factors to include. District of residence was a design variable and hence included in all models. Also, we eliminated redundant variables, e.g., because maternal education was highly correlated with maternal Raven's score and the HOME score, it was not included in the final models. Separate regression models were built for maternal and cord PCB concentrations. For interpretation of the regression model results, we took each beta coefficient, and back-calculated to obtain the predicted difference in BSID scores comparing the 25^th ^and 75^th ^percentile values, using the regression model: E(Y) = a *log_e _X, where X is PCB concentration, E(Y) is the expectation of the Bayley score, and a is the beta coefficient.

The SAS statistical package was used for analysis (Version 9.1, SAS Institute, Cary, NC).

## Results

Table 1 describes characteristics of two sub-populations with complete BSID scores and chemical measurements from maternal (n = 760) or cord (n = 258) serum samples, overall as well as by district. Characteristics were very similar between these two subsets, including maternal age, education and smoking history. However, in the group with cord PCB levels measured, there were slightly more female babies and more mothers who consumed alcohol during either the pregnancy or the three months before conception, in comparison with the larger group having maternal PCB measurements. By district, Svidnik had more maternal alcohol consumption history than Michalovce. Concentrations of the maternal and cord PCBs overall, as well as by district, are shown in Table 2. After lipid adjustment, the concentrations from the cord sera were slightly lower than those from the maternal sera (p < 0.0001), however the differences were greater for mono-ortho-substituted PCB congeners, for which the overall median of cord levels was about half the median concentration in the mothers. Overall, Svidnik had lower levels of PCB exposure compared to Michalovce, however, distributions overlapped considerably.

**Table 1:** Characteristics of the study groups in two districts of eastern Slovakia, 2002-2004.

Characteristics	Mother (N = 760)			Cord (N = 258)		
	
	% (n)			% (n)		
	Total	Michalovce	Svidnik	Total	Michalovce	Svidnik
	(n = 760)	73.5(559)	26.5(201)	(n = 258)	70.2(181)	29.8(77)
**Maternal age**						
< 19	2.6 (20)	2.6 (15)	2.5 (5)	2.3 (6)	2.8(5)	1.3(1)
< 29	70.0(532)	71.6 (400)	65.7(132)	70.2 (181)	71.8(130)	66.2(51)
29 +	27.4(208)	25.8 (144)	31.8 (64)	27.5 (71)	25.4 (46)	32.5(25)
**Maternal education**						
Basic schooling	18.4 (140)	21.3(119)	10.5(21)	18.2 (47)	21.5 (39)	10.4 (8)
High school without graduation	27.4 (208)	27.4(153)	27.4(55)	26.0 (67)	26.5 (48)	26.7 (19)
High school with graduation	46.7 (355)	45.3(253)	50.8(102)	48.8(126)	45.9 (83)	55.8 (43)
More than College/University	7.0 (53)	5.5(31)	10.9(22)	6.6 (17)	5.5 (10)	9.1 (7)
Missing	0.5 (4)	0.5(3)	0.5(1)	0.4 (1)	0.6(1)	-
**Sex of child**						
Male	51.3 (390)	51.3 (287)	51.2(103)	47.3(122)	49.7 (90)	41.6 (32)
Female	48.7 (370)	48.7(272)	48.8(98)	52.7(136)	50.3 (91)	58.4 (45)
**Ethnicity**						
Slovakian/other eastern European	79.2 (602)	77.3(432)	84.6(170)	79.8 (206)	77.6(140)	85.7 (66)
Romani	18.6 (141)	19.7(110)	15.4(31)	17.1 (44)	18.2 (33)	14.3 (11)
Missing	2.2 (17)	3.0(17)	-	3.1 (8)	4.4 (8)	-
**Marital status**						
Married or living with partner	91.6 (696)	90.0(503)	96.0(193)	91.4 (236)	89.5 (162)	96.1 (74)
Never married	5.5 (42)	6.1(34)	4.0(8)	4.7 (12)	5.5 (10)	2.6 (2)
Divorced/widowed	2.4 (18)	3.2(18)	-	2.7 (7)	3.3 (6)	1.3 (1)
Missing	0.5 (4)	0.7(4)	-	1.2 (3)	1.7(3)	-
**Maternal smoking**						
No	62.8 (477)	61.4 (343)	66.7(134)	61.2 (158)	56.9 (103)	71.4 (55)
Yes	34.8 (265)	35.6 (199)	32.8 (66)	35.7 (92)	38.7(70)	28.6 (22)
Missing	2.4 (18)	3.0 (17)	0.5 (1)	3.1 (8)	4.4 (8)	-
**Maternal Alcohol consumption**						
No	69.0 (525)	77.6 (434)	45.3 (91)	59.3 (153)	65.8 (119)	44.2 (34)
Yes	28.6 (217)	19.3 (108)	54.2(109)	37.6 (97)	29.8 (54)	55.8(43)
Missing	2.4 (18)	3.1(17)	0.5(1)	3.1 (8)	4.4 (8)	-
**Parity**						
0	38.4 (292)	39.0(218)	36.8(74)	38.4 (99)	40.9(74)	32.5 (25)
1	34.7 (264)	35.2(197)	33.3(67)	31.4 (81)	29.8 (54)	35.1(27)
2	17.9 (136)	17.5(98)	18.9(38)	20.5 (53)	21.0 (38)	19.5 (15)
3	8.6 (65)	7.7(43)	11.0(22)	8.5 (22)	6.6 (12)	13.0 (10)
4	0.3 (2)	0.4(2)	-	0.8 (2)	1.1(2)	-
Missing	0.1 (1)	0.2(2)	-	0.4 (1)	0.6 (1)	-
**Maternal hyper- or hypothyroidism history**						
No	94.7 (720)	94.5(528)	95.5(192)	93.4 (241)	93.9 (170)	92.2 (71)
Yes	3.1 (23)	2.5(14)	4.5 (9)	3.5 (9)	1.7 (3)	7.8(6)
Missing	2.2 (17)	3.0 (17)	-	3.1 (8)	4.4(8)	-
**Maternal diabetes history**						
No	96.6 (734)	95.7 (535)	99.0(199)	95.3 (246)	94.5(171)	97.4 (75)
Yes	1.2 (9)	1.3 (7)	1.0 (2)	1.6 (4)	1.1(2)	2.6 (2)
Missing	2.2 (17)	3.0(17)	-	3.1 (8)	4.4(8)	-
**Raven's score**						
Mean (SD)	41.0(12.9)	40.6 (13.7)	42.2 (10.4)	40.5 (13.3)	39.4 (14.1)	43.8 (11.1)
**MDI**						
Mean (SD)	93.8 (13.4)	93.1(13.5)	95.7 (13.0)	91.0(13.5)	89.7 (13.7)	94.1 (12.7)
**PDI**						
Mean (SD)	99.8 (15.1)	103.1(14.3)	90.6(13.6)	98.6 15.4)	100.9 (15.5)	93.0 (13.7)

**Table 2:** Distributions of maternal and cord PCBs concentration (ng/mg lipids) in two districts of eastern Slovakia, 2002-2004.

		**5**^**th**^	25th	50th	75th	95th	Mean	SD
**Maternal sera**								

∑ 4 Major PCBs^† ^(non-dioxin-like PCBs)	Total (n = 760)	0.159	0.280	0.414	0.659	1.583	0.590	0.663
	Michalovce (n = 559)	0.197	0.346	0.502	0.751	1.815	0.685	0.718
	Svidnik (n = 201)	0.122	0.193	0.254	0.356	0.660	0.324	0.366
∑ Dioxin-like mono-ortho PCBs^†^	Total	0.005	0.012	0.021	0.036	0.093	0.032	0.047
	Michalovce	0.005	0.015	0.025	0.041	0.106	0.037	0.052
	Svidnik	0.003	0.008	0.014	0.019	0.038	0.017	0.018
∑Anti-estrogenic PCBs^†^	Total	0.108	0.194	0.284	0.458	1.093	0.410	0.470
	Michalovce	0.136	0.237	0.347	0.523	1.255	0.478	0.511
	Svidnik	0.085	0.131	0.174	0.248	0.424	0.222	0.249
**Cord sera**								

∑ 4 Major PCBs (non-dioxin-like PCBs)	Total (n = 258)	0.096	0.228	0.380	0.666	1.477	0.548	0.537
	Michalovce (n = 181)	0.177	0.332	0.493	0.807	1.584	0.682	0.584
	Svidnik (n = 77)	0.067	0.139	0.216	0.280	0.555	0.231	0.142
∑ Dioxin-like -substituted PCBs	Total	0.003	0.005	0.011	0.028	0.084	0.022	0.030
	Michalovce	0.004	0.007	0.017	0.034	0.089	0.028	0.034
	Svidnik	0.003	0.004	0.005	0.008	0.031	0.008	0.008
∑Anti-estrogenic PCBs	Total	0.062	0.154	0.246	0.441	0.985	0.372	0.384
	Michalovce	0.120	0.225	0.343	0.547	1.053	0.465	0.421
	Svidnik	0.043	0.085	0.145	0.188	0.375	0.152	0.097

^† ^∑ 4 major PCBs (non-dioxin-like PCBs), sum of PCB #138, #153, #170 and #180; ∑ Dioxin-like PCBs, sum of PCB #118, and #156; ∑ Anti-estrogenic PCBs, sum of PCB #138, #156, #170, and #180

Table 3 presents the summary of estimated coefficients, their standard errors, and *p*-values for exposure variables in multiple linear regression models predicting Bayley scales (MDI and PDI) after adjusting for residential district, HOME scores, sex, and Raven scores of mothers. Neither maternal nor cord di-ortho-substituted PCBs (#138, #153, #170, and #180) were associated with the Bayley scores, and there was no association between the group of anti-estrogenic PCBs (#138, #156, #170, and #180) and the Bayley scores. In contrast, concentrations of dioxin-like mono-ortho PCBs (sum of #118 and #156) were associated with significantly lower scores on both the MDI and PDI Scales, using either maternal or cord PCB measurements. Findings for the mono-ortho-substituted PCBs were similar for maternal and cord PCBs (Intraclass correlation (ICC):0.88), particularly in relation to the PDI. For the non-dioxin-like, di-ortho-substituted PCB congeners (ICC: 0.95), an association was present for the PDI only and was stronger with cord than with maternal PCB concentrations.

**Table 3:** Estimated coefficients with *p*-values of PCBs and their subsets in relation to BISD at age of16 months, 2002-2004^†^.

Log transformed	Maternal sera (N = 760)				Cord sera (N = 258)			
Exposure Variables	MDI		PDI		MDI		PDI	
	β	SE	β	SE	β	SE	β	SE
4 major PCBs^‡ ^(∑non-dioxin-like PCBs)	-0.39	0.62	-0.59	0.73	-1.02	0.94	-1.95*	1.13
∑ Dioxin-like mono-ortho PCBs ^‡^	-1.60***	0.45	-1.26**	0.53	-1.40*	0.72	-1.99**	0.87
∑Anti-estrogenic PCBs^‡^	-0.47	0.61	-0.58	0.72	-0.93	0.94	-1.85	1.13
PCB 118	-1.17***	0.39	-1.04**	0.46	-1.44**	0.64	-2.04***	0.77
PCB 138	-0.30	0.59	-0.53	0.70	-1.35	0.91	-2.27**	1.09
PCB 153	-0.33	0.62	-0.63	0.73	-1.22	0.94	-2.14*	1.14
PCB 156	-1.46***	0.43	-1.02**	0.50	-0.50	0.71	-0.63	0.86
PCB 170	-0.54	0.60	-0.58	0.71	-0.62	0.77	-1.90*	0.94
PCB 180	-0.36	0.61	-0.47	0.72	-0.55	0.94	-1.26	1.13

*P < 0.1; **P < 0.05; *** *P *< 0.01; Regression parameter coefficients represent score change in PDI and MDI for a one-unit change in log_e _[exposure concentration ng/mg lipid].

^‡ ^∑4 major PCBs (non-dioxin-like PCBs), sum of PCB #138, #153, #170 and #180; ∑ Dioxin-like mono-ortho PCBs, sum of PCB #118, and #156; ∑Anti-estrogenic PCBs, sum of PCB #138, #156, #170, and #180

^† ^After adjusting for district, HOME, sex, Raven score from multiple linear regression models.

Additionally, each of the 6 major PCB congeners (#118, #138, #153, #156, #170, and #180) was tested in separate regression models, and maternal and cord concentrations of PCB #118 were most strongly associated with reduced BSID.

Table 4 shows separate final full multiple regression models with confounders, namely, district, HOME score, Raven's score, and sex of the babies, predicting MDI and PDI from the mono-ortho-substituted PCBs in maternal and cord sera. The interpretation of these coefficients is that when the maternal concentration of the sum of the two mono-ortho dioxin-like PCBs is increased from the 25^th ^to 75^th ^percentile in this study (0.012 to 0.036 ng/mg lipids), MDI (*p *< 0.001) and PDI (*p *= 0.02) are reduced by approximately -1.8 and -1.4 points, respectively. Similarly, the inter-quartile increase of the sum of these same PCBs in cord serum (0.005 to 0.028 ng/mg lipids) was significantly associated with -3.4 point reduction in PDI (*p *= 0.02), but marginally with -2.4 points in MDI (*p *= 0.05). Regarding other covariates, girls had a higher MDI and PDI in comparison with boys. As expected, higher HOME and maternal Raven scores were associated with higher MDI and PDI.

**Table 4:** Final full multiple linear regression models of dioxin-like mono-ortho PCBs *.

Maternal sera (n = 760)						
	**MDI**			**PDI**		

**Variables**	**Beta**	**SE**	***p *value**	**Beta**	**SE**	***p *value**

Dioxin-like mono-ortho PCBs (natural log transformed: ng/mg lipids)	-1.60	0.45	< 0.001	-1.26	0.53	0.02
District: Michalovce vs. Svidnik	-4.12	1.01	< 0.0001	-17.22	1.19	<0.0001
HOME score	1.23	0.10	< 0.0001	0.80	0.12	< 0.0001
Raven score	0.24	0.04	< 0.0001	0.25	0.04	< 0.0001
SEX: male vs. female	3.27	0.78	< 0.0001	3.40	0.92	0.0002

**Cord sera (n = 258)**						

	**MDI**			**PDI**		

**Variables**	**Beta**	**SE**	***p *value**	**Beta**	**SE**	***p *value**

Dioxin-like mono-ortho PCBs (natural log transformed: ng/mg lipids)	-1.40	0.72	0.05	-1.99	0.87	0.02
District: Michalovce vs. Svidnik	-4.15	1.77	0.02	-15.0	2.13	< 0.0001
HOME score	1.12	0.16	< 0.0001	0.64	0.19	<0.001
Raven score	0.20	0.06	0.001	0.38	0.08	< 0.0001
SEX: male vs. female	4.04	1.77	0.02	3.51	1.61	0.03

* This final model provides an estimate of the effect of dioxin-like mono-ortho PCBs in maternal and cord sera on the Bayley scales (MDI and PDI), adjusting for district, HOME score, sex, maternal Raven's score.

## Discussion

We observed higher concentrations of two mono-ortho PCBs in maternal sera to be associated with decrements in neurodevelopment at 16 months of age. Several lab-based studies reported alteration of dopamine (DA) levels, changes of behavior, or a reduction in long-term potentiation (LTP), regarded as the cellular and molecular mechanisms that are linked to some forms of learning and memory, by coplanar dioxin-like PCBs such as congener #77 or #126 [60-64]. However, other studies in rats reported that the coplanar dioxin-like PCB #126 was not associated with deficits in attention and behavioral performances [65,66].

Regarding epidemiological studies, although the birth cohort study from the Netherlands found an association of coplanar and mono-ortho dioxin-like PCBs (# 77, 126, 169, 105, 118, 156) in breast-milk collected two weeks after delivery with suboptimality at birth, their findings were null at 7, and 18 months for the PDI and MDI except they found a significant association with decreased PDI at 3 months [14,67,68].

Reports from Germany indicated reduced MDI at 7, 18 and 30 months in relation to prenatal PCBs (the sum of PCB #138, #153, and #180), although the 18-month scores were somewhat less precise (*p *= 0.06) as compared with the other time points [13,15]. Our study didn't show associations with either non-dioxin like PCBs or the sum of PCB #138, #153, and #180 even though the concentration of the sum of PCB #138, #153, and #180 was higher than that of German study (1.16 vs 0.55 ng/ml) [15]. It is not certain if our inconsistent findings with the German study were from differences between the two studies in other nonquantitated PCBs or in other chemical compounds that correlated with the measured non-dioxin like PCBs, or for other methodologic reasons.

A study with 6-month-old children in Japan [69] reported no statistically significant association between prenatal non-dioxin like PCBs (#170, #180) and the Bayley scores while some dioxin-like PCBs (#118,#126, #157, and #189) showed suggestive associations with reduced PDI (*p *< 0.1) but not MDI. Compared to our levels of maternal PCBs, median concentrations in this study of PCB #118, #156, #170, and #180 were lower by a range of approximately 2 to 13 fold. Some research on non-dioxin-like PCBs [24,70] suggests that they tend to show more potency for neurotoxicity than dioxin-like PCBs through various mechanisms: interference with protein kinase C(PKC) [70,71], changes in DA levels [23], and apoptosis [72]. In contrast to some other epidemiological or lab-based studies [13,15,24,70], we found the evidence was weaker for a relationship between neurodevelopment and prenatal exposures to di-ortho-substituted PCBs, although a suggestive association was observed with PDI (β = -.195, *p *= 0.09) from these compounds measured in cord, but not in maternal samples (Table 3).

Both mono-ortho and di-ortho substituted PCBs can affect nervous system tissues, but these effects are possibly mediated by different pathways [73]. In a related publication, we evaluated the hydroxylated metabolites of PCBs in relation to the scores on the Bayley Scales and reported that 4-OH-PCB107 was the only metabolite that showed any relationship to neurodevelopment at 16 months [74]. Both maternal and cord serum concentrations of 4-OH-PCB107 were associated with deficits on the MDI, and cord levels were also associated with deficits on the PDI. As 4-OH-PCB107 is the metabolite of the two congeners PCB #105 and PCB #118, both being mono-ortho substituted, the two investigations present a consistent signal: increased prenatal exposures to both the parent mono-ortho PCB compounds and the hydroxylated metabolites of those mono-ortho PCBs occur in association with lower neurobehavioral scores. The question therefore arises: through what mechanism might one or more of these compounds, but not the di-ortho substituted congeners and corresponding metabolites measured in our cohort, act?

Mechanisms hypothesized to underlie PCB neurotoxicity have included thyroid disruption [75], interference with sex steroids either as agonists or antagonists [64,76], and Ah-receptor activity. Thyroid hormones are critical to neuronal proliferation, migration, synaptogenesis, and brain myelination [77]. It has long been hypothesized that one mechanism for neurodevelopmental toxicity of PCBs is through disruption of thyroid hormones [78], and more recently, hydroxylated metabolites have been implicated in this mechanism because of greater transplacental transfer than the parent compounds [79], high affinity for transthyretin, a transport protein, and accumulation in fetal tissues, particularly 4-OH-CB107 [80-82]. A recent *in vitro *study showed that PCB 105 and/or 118 act as thyroid hormone receptor agonists, but only in the presence of PCB 126 induction of CYP1A1 [83]. The lack of a similar effect on thyroid receptors from PCB #138 and #153 conforms with our results, supporting plausibility of this mechanism for 4-OH-CB107, the common metabolite of two mono-ortho PCB #105 and #118, in impairment of neurodevelopment. Disruption of thyroid hormones may itself operate via altered cell signaling and/or neurotransmitters [78].

We did not find significant associations of neurodevelopmental scores with anti-estrogenic PCBs (sum of #138, 156, 170 and 180), but did not evaluate possible roles of PCB metabolites with regard to estrogenic and/or anti-estrogenic activities in this paper. As mentioned above, in our previous work examining six hydroxylated metabolites [74], only 4-OH-CB-107 was significantly associated with decreased Bayley scores. Interestingly, an *in vitro *study that examined inhibition of an estrogen inactivator by OH-PCBs and subsequent increases in estrogen levels at the target tissue, reported 4-OH-CB-107 to be one of strongest inhibitors of the human estrogen sulfotransferase (hEST), an enzyme that catalyzes the sulfation and inactivation of estrogens. The hEST activity was 1.5 to 2 orders of magnitude greater than that observed for 4-OH-CB-146 or 4'-OH-CB-172 [84], metabolites of PCB 138, 153, and PCB 170, 180 respectively. Thus, despite previous mixed reports of anti-estrogenicity [85] or estrogenicity [86] for 4-OH-CB-107, (as well as similar observations for estrogenic [51] and anti-estrogenic [87] activity for the parent PCB 118), a possible mechanism of neurodevelopmental toxicity is through hEST induction of estrogenic activity, rather than binding directly to the estrogen receptor. This could provide a coherent explanation for our observations linking deficits in neurodevelopment to PCB 118 and/or its metabolite, 4-OH-CB-107, but not to the di-ortho congeners or their OH-metabolites.

Ah-receptor activity as shown for non-ortho substituted PCBs, is another plausible mechanism for the associations we observed. On the one hand, dioxin-like activity of mono-ortho PCBs occurs only at much higher concentrations than that of coplanar PCBs or of 2,3,7,8-TCDD; on the other, congeners #118 and #156 are present, in virtually every human population studied, at levels from 4 to 5 orders of magnitude greater than 2,3,7,8-TCDD itself (e.g., in U.S., European, and Japanese studies [88-91]). Because of the exceedingly low levels of the coplanar PCBs, furans, 2,3,7,8-TCDD and other dioxins and the high cost to quantitate them, we were unable to measure these compounds in cord and maternal sera. However, in adult women aged 20-39 from the same population, i.e., the same two Slovak towns, we have calculated TEQs using the WHO 2005 revised TEFs for mono-ortho PCBs (0.00003) for the sum of PCB #118 and #156 (geometric mean TEQ, 1.36 pg/g lipid), and they are over four times higher than the TEQs for 2,3,7,8-TCDD itself (0.32 pg/g lipid). In other words, the lower toxicity was more than compensated by the far higher abundance, supporting relevance of dioxin-like activity from mono-ortho PCBs in this population.

A cautionary note is in order, however: TEFs are calculated based on intake and are not considered applicable to circulating levels in human tissues [17,92]. The WHO 2005 report [17] calls for studies that would provide TEFs for tissue concentrations, but at present this research has yet to be conducted. Whether the actual TEQ in this study is higher or lower than we calculated remains unclear, but the shorter half-life of PCB #118 in comparison with that of 2,3,7,8-TCDD indicates relatively greater intake would be required to maintain blood levels, while the half-life of PCB #156 may be similar to that of 2,3,7,8-TCDD [93]. The TEQs for intake of the two mono-ortho PCBs together would therefore likely be larger than what we have calculated based on circulating concentrations. Also notable is a recent effort to develop a Neurotoxic Equivalence Scheme that would take into account multiple mechanisms [94]. Another concern about the calculation of dioxin-like potencies in epidemiologic studies relates to the difficulty in extrapolating from rat to human, given that large differences in AhR-mediated gene expression and EROD activity are observed even comparing rats with mice [95].

Further concerns have focused on the presence of impurities in test samples, specifically 2,3,7,8-substituted polychlorinated dibenzo-dioxins (PCDDs), -furans (PCDFs), or PCB 126 [17]. Our inability to measure trace quantities of these compounds precludes drawing inferences about the role of impurities, although other data (personal communication with Dr. Kocan, 2009) from women of roughly the same age range in these two towns demonstrate low correlations of the sum of PCB #118+ #156 with 2,3,7,8-TCDD (0.016, *p *= 0.94) and with coplanar PCBs (0.26, *p *= 0.23) and negative correlations with PCDDs (-0.31, *p *= 0.15) and PCDFs (-0.29, *p *= 0.17). Thus, the associations we observed for neurocognitive developmental deficits with the sum of the two most abundant mono-ortho PCBs are unlikely to be a result of confounding from these other compounds.

The subset with cord PCBs measured had similar sociodemographic characteristics to the larger group with maternal PCBs, and sensitivity analyses did not indicate selection bias. Since the mono-ortho-substituted PCBs were present at relatively lower concentrations in cord than maternal serum, whereas di-ortho-substituted PCBs were at similar levels, these findings may indicate that PCBs measured in the cord are more potent, and/or a better marker of what reaches the target CNS tissue in the fetus.

A large refusal rate (47 percent) for participation and attrition (29 percent) of the follow-up represented a study limitation. The eligibility of those who refused is unknown, but given our exclusions based on residence in the district, parity, and age, a substantial proportion of the refusals may have been ineligible. Comparing those lost to follow-up vs. those who stayed in the study, lipid adjusted levels of 4 major PCBs (#138, #153, #170, and #180) were not different (0.57, and 0.59 ng/mg lipids, respectively) implying that the study losses were not related with exposure levels. On the other hand, there were some differences in regard to socio-demographics (data not shown): those lost to follow-up included more Romani (28.0 vs. 18.4 percent), more Svidnik residents (37.0 vs. 26.7 percent), more participants with basic schooling only (28.0 vs. 18.6 percent), and more women of low parity (49.2 vs. 38.3 percent) compared to those who returned for the 16 month follow-up visit. BSID scores for those lost to follow-up were, by definition, not obtained in our study. Nevertheless, our final model included adjustments for HOME and Raven's scores, which relate to the infant's nurturing environment at home and the mother's IQ respectively. If missingness was at random within any given level of the adjustment variables, bias from non-random loss of subjects would be less likely.

The means of MDI for the two psychologists were similar (93.1 for Michalovce, n = 559; 95.7 for Svidnik, n = 201), but lower than the mean scores of 100 in the populations for which they are standardized. Because the BSID has not been normed in the Slovak language or culture, some cross-cultural or language differences might have played a role in the lower MDI scores in our study [96]. Our research objective was to examine possible associations between prenatal PCBs and BSID scores within this study cohort. Therefore, while comparisons of BSID scores with other populations might not be valid, the absolute scores are not the focus of our investigation.

**Figure 1:**
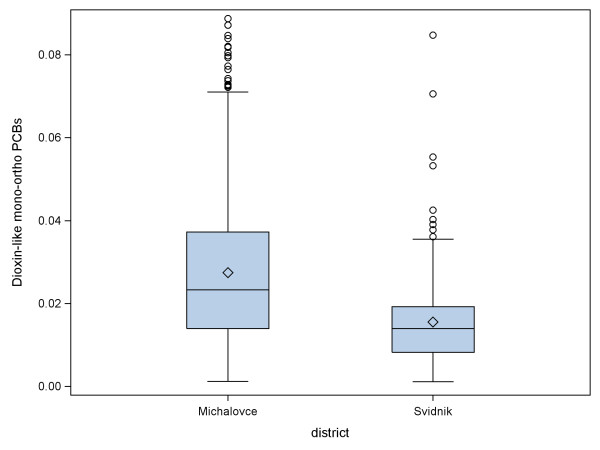
**Maternal dioxin-like mono-ortho-substituted PCB concentrations (ng/mg lipids) by district**.

For reliability of the BSID scores, good agreement on the MDI (ICC: 0.84) between raters was obtained. However, we were not able to evaluate reliability on the PDI because the child frequently moved out of range of the video camera during the test. Also, we observed a large difference in mean scores of PDI, approximately 10 points, between two districts. This suggests the possibility of poor reliability across raters, and/or some other unmeasured variables related to district that might have resulted in the substantial difference in PDI. Therefore, we also examined the effects of maternal dioxin-like-PCBs in each district separately (results not shown). In Michalovce, where the psychologist is more experienced, we still observed significant associations, with similar magnitude as for the entire sample, between mono-ortho substituted PCBs and the Bayley scores; in Svidnik, the district with a less experienced psychologist, we did not find any association with maternal mono-ortho substituted PCBs. Overall, PCB exposure distributions substantially overlapped for these two districts, however, mean PCB levels were significantly higher in Michalovce (which had a longer right tail) than in Svidnik (Figure 1). Adjustment for district is required because it was a design variable, but would not have constituted over-adjustment for PCB concentrations.

As mercury and lead are well-known neurotoxins, we considered the possibility of confounding. However, in a sub-sample of the cohort, correlations between these heavy metals and dioxin-like PCBs didn't exist (correlation coefficients were either negative or less than 0.03, and none of them were significant). Although fish can be a major source of methyl mercury, in other work from our Slovak birth cohort, mothers' fish consumption was very low, contributing a negligible amount of fat to the diet, and hence would not be expected to be an important source of PCBs [97].

Multiple comparisons can be a problem in the study because we tested 36 various combinations of PCBs and outcomes. While some of our results could have been due to chance, it is not possible statistically to determine which ones are just random variations and which represent true underlying associations. P-values are based on the assumption of the universal null hypothesis being correct, whereas *a priori *knowledge motivated several of the hypotheses we examined, specifically those related to different congener classes; in this situation, multiple comparisons can be justifiable as part of a careful and thorough evaluation of patterns of associations [98]. Notably, our significant results were not sporadic, as would be expected if they were chance findings, but rather showed a pattern whereby dioxin-like congeners showed the strongest associations in all analyses: cord or maternal serum, MDI or PDI. Furthermore, the pattern is upheld by our analysis of hydroxy-metabolites of PCBs: of the six that we evaluated, the only one associated with neurobehavioral deficits was 4-OH-PCB107, the metabolite for PCB #118 and another mono-ortho congener [74].

Several strengths are worth emphasizing: the large sample size; direct exposure assessments of prenatal PCBs rather than using a surrogate such as breast-milk or fish consumption during pregnancy; good quality control of laboratory measurements. Unfortunately, not many previous epidemiological studies have examined functionally-defined subsets of PCB congeners, and fewer still have done so in relation to neurodevelopment. Also, we were able to measure only a limited roster and some were present at very low levels relative to our limits for quantitation, which prevented a more definitive assessment of associations with regard to neurodevelopment in children.

A change in a few points on the Bayley scales may seem clinically unimportant for an individual child. However, beginning with studies from the population level, it can be predicted that a slight shift in the mean performance in the adverse direction can result in a substantial increase in the number of children who are in the subnormal range in the clinical setting [99].

## Conclusion

Based on the results herein, the high levels of mono-ortho PCBs, and the literature on dioxin-like activity in relation to neurodevelopment, we conclude that the associations of prenatal dioxin-like mono-ortho PCBs, in either maternal or cord serum, with decreased performance in both the cognitive and motor domains based on BSID scores could plausibly be causal. However, a suggestive association of di-ortho-substituted PCBs with decreased motor development was found in cord but not maternal serum, and we did not find statistically significant associations with anti-estrogenic PCBs.

## Abbreviations

(BSID): The Bayley Scales of Infant Development; (MDI): Mental Development Index; (Polychlorinated biphenyls): PCBs; (PDI): Psychomotor Development Index.

## Competing interests

The authors declare that they have no competing interests.

## Authors' contributions

Author HYP wrote the manuscript and conducted statistical analyses. Author ES supervised the field activities of cognitive assessment. Author AK and BD were responsible for chemical analysis including quality control and quality assurance. Authors IHP and TT designed and directed the whole study. Additionally, IHP contributed significantly to the writing and revising of the paper. All authors have read and approved the final manuscript.
